# Incidentally Discovered Giant Benign Ovarian Serous Cystadenoma in Elective Bariatric Surgery

**DOI:** 10.7759/cureus.60807

**Published:** 2024-05-21

**Authors:** Lucy P Kelly, Roshni B Patel, Danuel Laan

**Affiliations:** 1 Department of Research, Alabama College of Osteopathic Medicine, Dothan, USA; 2 Department of Surgery, USA Health Providence Hospital, Mobile, USA

**Keywords:** ovarian tumours, bariatric surgery morbid obesity, ovarian cystectomy, giant ovarian tumors, asymptomatic mass, weight loss and obesity, ovarian serous cystadenoma

## Abstract

Ovarian cystadenomas are benign epithelial neoplasms, many of which are of the serous subtype. Most patients present with symptoms such as abdominal pain, bloating, and bladder issues. This patient, who had a BMI of 45, presented with a giant ovarian serous cystadenoma identified during an elective bariatric surgery; interestingly, she was completely asymptomatic at the time of discovery. A large, predominantly cystic pelvic mass with internal septations and soft tissue components, suspicious for ovarian neoplasm, was discovered on a CT abdomen and pelvis with IV contrast. She underwent an exploratory laparotomy with complete resection, right oophorectomy, and ovarian cystectomy. Her postoperative pathology report revealed the mass to be a benign serous cystadenoma. This case serves as an example of how a massive tumor can potentially get overlooked for many years, only to be detected unintentionally in an asymptomatic patient. Healthcare quality is often negatively impacted by the inherent prejudice that many healthcare providers have toward their obese patients. Providers may mistakenly over-attribute a patient’s symptoms to their obesity, failing to effectively evaluate the patient’s concerns, which could lead to overlooking potentially harmful diagnoses. A comprehensive history and physical exam in all patients, especially those who are obese, is vital in ensuring timely diagnosis and management to improve patient outcomes.

## Introduction

Ovarian cystadenomas are benign epithelial neoplasms. Cystadenomas occur commonly as mucinous or serous types. Benign serous cystadenomas of the ovary make up 16% of all ovarian epithelial neoplasms and two-thirds of benign ovarian epithelial tumors [1]. Serous cystadenomas consist of cysts and papillae that are lined by either stratified or non-stratified cuboidal or columnar cells that appear like fallopian tube epithelium [1]. Adults of all ages are affected by this tumor, with the mean age falling between 40 and 60 years. In 10-20% of the cases, these tumors are bilateral [2]. Patients often present with clinical symptoms such as nonspecific abdominal or pelvic pain, bloating, nausea, and vomiting. These symptoms are usually related to the pressure that a large cyst can exert on the surrounding structures, meaning that giant cysts may cause more symptoms [3].

We present a case in which a patient was diagnosed with a benign ovarian serous cystadenoma, with dimensions measuring 16 cm x 13 cm x 16 cm. On average, serous cystadenomas are approximately 10 cm in diameter [1], making this particular patient’s case impressive since it is larger than normally observed. With this publication, we aim to report a unique case of a giant ovarian serous cystadenoma in an obese woman, who was unusually asymptomatic until the tumor was incidentally discovered during bariatric surgery.

## Case presentation

A 59-year-old Caucasian female presented to the bariatric surgery clinic in Mobile, Alabama, for a weight-loss surgery consultation in January 2023. She stated that she had tried various methods of weight loss including multiple diet programs such as low-calorie, intermittent fasting, Nutrisystem, and Weight Watchers. Also, she tried numerous exercise programs. The patient lost weight with every attempt, but always gained it back. She was 248 lb at this visit, which was the heaviest weight she had ever been. She had no complaints or discomfort otherwise. She had a past medical history of morbid obesity (BMI of 45), obstructive sleep apnea (OSA), hyperlipidemia, gastroesophageal reflux disease (GERD) with known hiatal hernia, hyperparathyroidism, and metabolic syndrome. She had a pertinent history of two term pregnancies and total abdominal hysterectomy in 2009. Her current medications include famotidine 40 mg daily, pantoprazole 40 mg daily, and phenteramine 37.5 mg daily. Family history was significant for an unspecified malignancy in her sister. She had a 33 pack-year smoking history, but she quit 10 years ago. 

**Figure 1:**
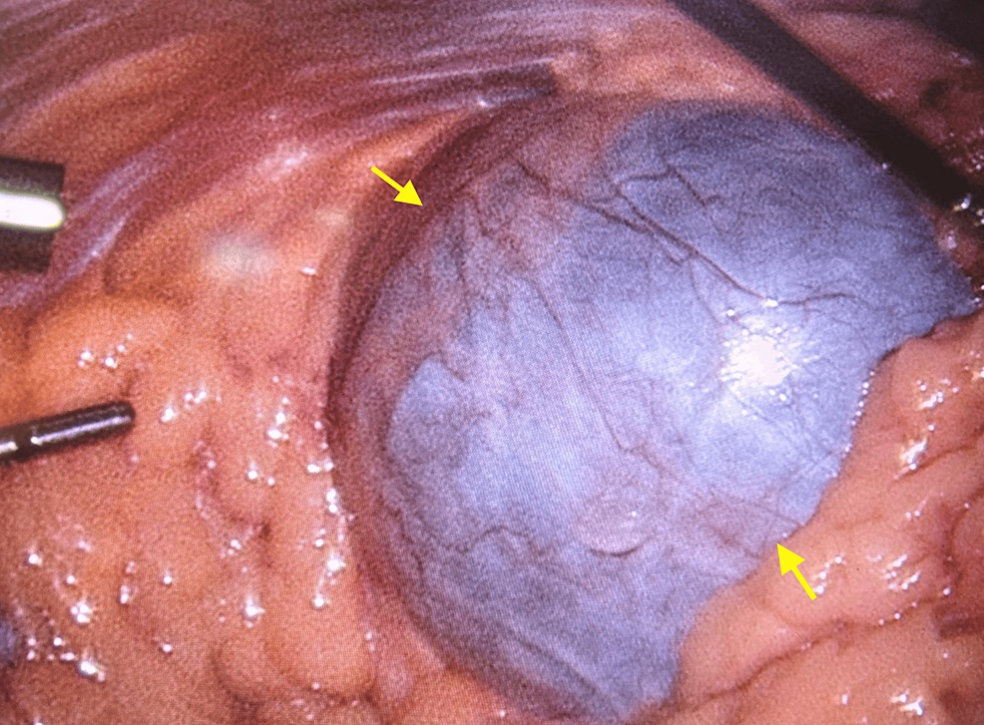
Large pelvic mass, the borders of which are indicated by yellow arrows, discovered during sleeve gastrectomy and hiatal hernia repair.

**Figure 2:**
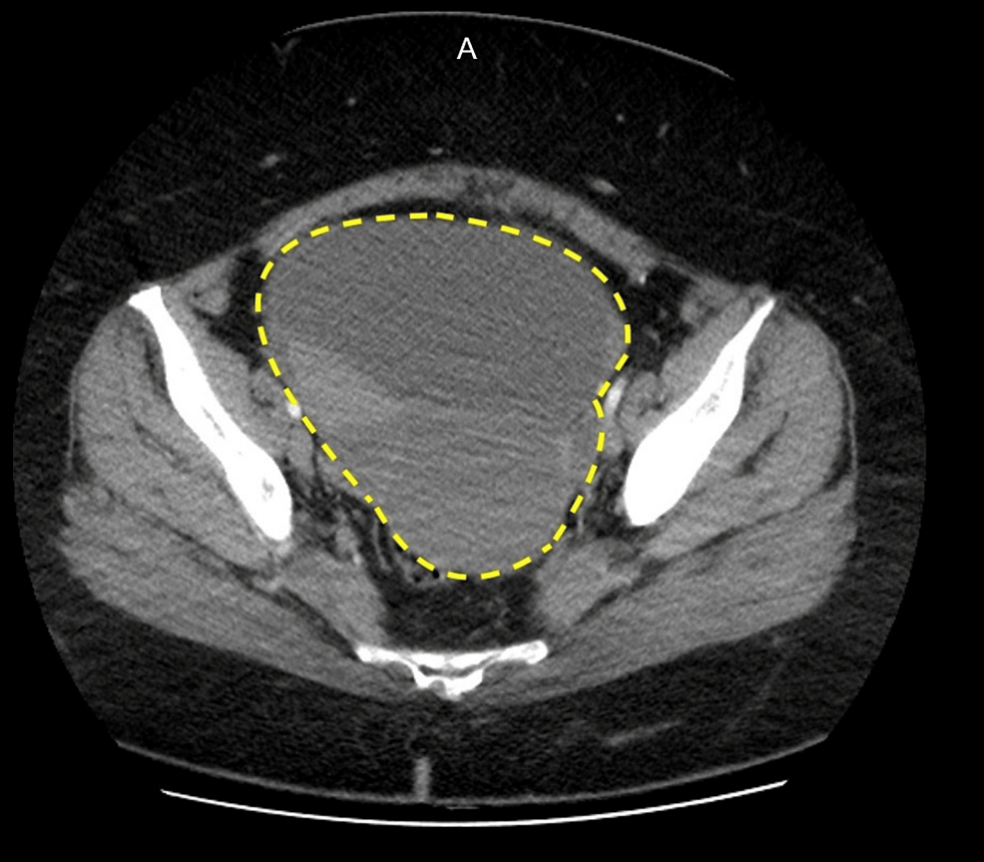
Transverse contrast-enhanced abdominal and pelvic CT showing a large cystic pelvic mass, outlined by a dotted yellow line.

**Figure 3:**
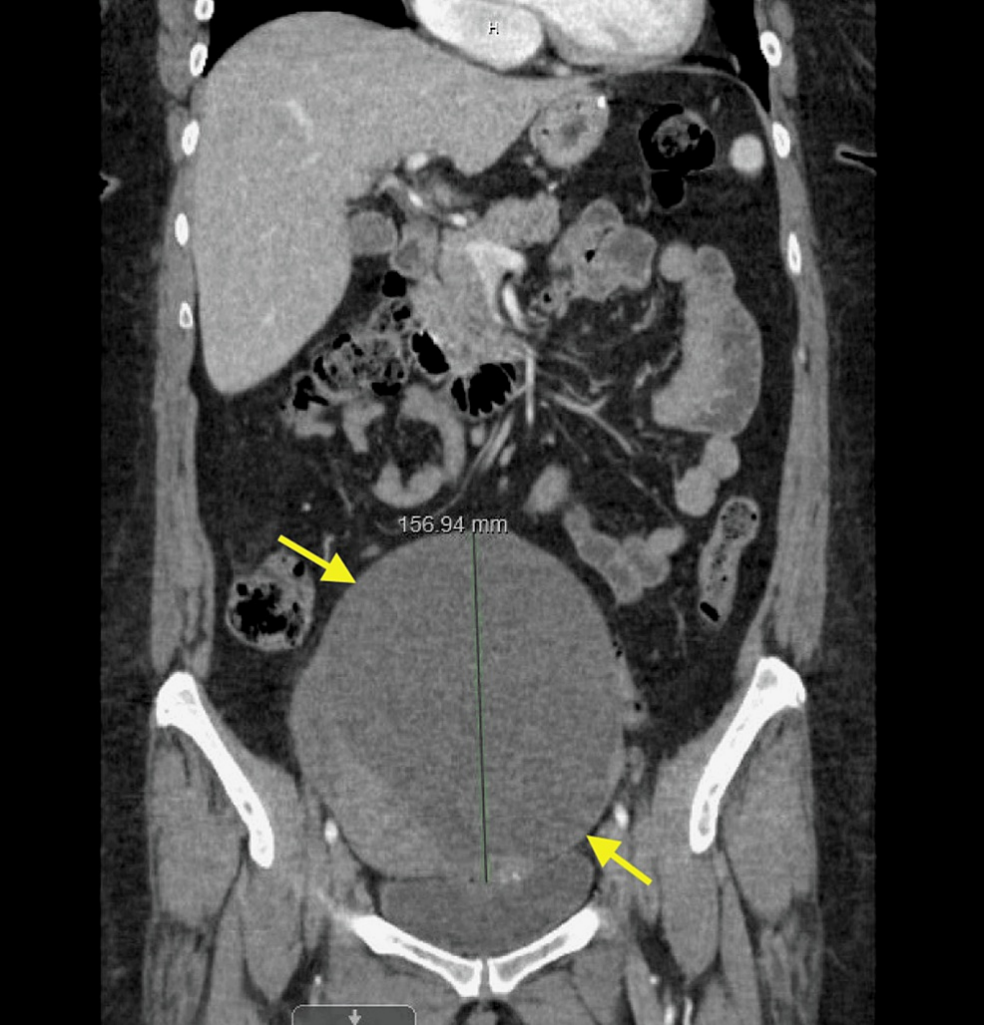
Coronal contrast-enhanced abdominal and pelvic CT revealing a large cystic pelvic mass (yellow arrows).

She underwent a sleeve gastrectomy with hiatal hernia repair in July 2023. During the procedure, a large pelvic mass was incidentally discovered. It did not appear to be growing into any adjacent structures (Figure 1). Sleeve gastrectomy was deemed safe to proceed with, and she tolerated it well with no complications. She was referred for follow-up with gynecological oncology as this mass needed resection. A CT abdomen and pelvis with IV contrast was done, revealing a large (16 cm x 13 cm x 16 cm) predominantly cystic pelvic mass with internal septations and soft tissue components, suspicious for ovarian neoplasm (Figures 2, 3). The mass was causing mild hydronephrosis and ureteral dilatation secondary to partial obstruction. Postoperative changes from the gastric sleeve surgery were also seen on imaging.

She was then scheduled to have an exploratory laparotomy. Prior to the surgery, a physical exam of the patient was performed by the gynecological oncologist. Abdomen was obese and non-distended, soft, and nontender with no masses. On pelvic exam, the pelvic external mucosal was clear while the vaginal vault was clear and atrophic. No vaginal discharge or bleeding was observed. On the bimanual exam, there was a large, soft mass to the right of the midline that was soft, fixed, and nontender. Urinalysis was also performed, which showed nothing of significance.

In September 2023, she underwent an exploratory laparotomy with complete resection, right oophorectomy, and ovarian cystectomy. The mass was fully resected with no complications. She tolerated the procedure well. Post-operative pathology report revealed benign serous cystadenoma.

## Discussion

Ovarian serous cystadenomas are benign epithelial neoplasms that vary in size and can reach massive measurements. These tumors affect a wide range of adults, with the mean age falling between 40 and 60 years. While these tumors are not rare, the average size is typically 10 cm. Generally, the larger an ovarian lesion is, the more likely that it is a neoplastic process requiring surgical intervention [4, 5]. In evaluating adnexal masses, obtaining a transabdominal or transvaginal ultrasound is the best first step. CT and MRI can then be used to better visualize the specific ovarian mass [6]. Patients with these masses can present with symptoms including nausea, vomiting, bladder issues, nonspecific abdominal pain, pelvic pain, and bloating [7]. It is vital to consider past medical history, smoking history, surgical history, family history, and tobacco use when assessing these patients in clinical scenarios. Our patient had several risk factors that may have contributed to the development of this benign tumor, including current obesity and a history of hysterectomy. Obesity at age 20, as well as recent obesity, is associated with an increased risk of benign serous ovarian tumors. Additionally, having a hysterectomy appears to put a patient at a greater risk of developing this tumor [8]. 

This case exemplifies the importance of a complete and thorough history and physical exam in morbidly obese patients, as their body habitus can make it difficult to detect these dangerous tumors. There are multiple points of care in which tumors like this could be detected on a physical exam, such as during a routine physical exam by a primary care physician or during a pelvic exam by an obstetrician/gynecologist. The patient’s history of obesity may have contributed to physicians missing the tumor throughout its disease progression. The quality of healthcare is often hindered by the prejudice that healthcare providers have toward people with obesity. While overweight and obese patients often have more comorbid conditions than normal-weight patients, they are known less well by their physicians. Additionally, physicians build significantly less rapport with obese individuals, further emphasizing that they may engage with these patients at an emotional distance [9]. Many providers view obesity as an avoidable risk factor that disrupts their ability to treat and prevent other diseases. They often over-attribute symptoms to obesity and fail to appropriately evaluate patient concerns, including gathering appropriate diagnostic testing or considering appropriate treatment options beyond encouraging weight loss. On the other hand, patients often feel embarrassed or unwelcomed at doctor’s offices, making them less likely to seek recommended screenings for some cancers. This leads to delayed care in these populations, which causes obese patients to present with these conditions at more advanced, difficult-to-treat stages [10]. The biases that physicians have toward those with obesity may have also led to this tumor getting missed for diagnosis.

Ovarian serous cystadenomas can malignantly transform into ovarian serous cystadenocarcinomas. Approximately 20%-25% of these tumors are malignant, highlighting the importance of proper screening and prompt diagnosis in the management of these diseases [11]. By surgically excising the tumor, the patient avoided possible negative health impacts in the future. Additionally, while this tumor was benign, its large size has the potential to cause mass effect to surrounding tissues and organs. Symptoms of mass effect include mild abdominal pressure and pain to more severe life-threatening complications, like cystic rupture, ovarian torsion, and hemorrhage [6]. For these reasons, early detection of these masses would prevent both malignancies and severe symptoms while they are more manageable.

## Conclusions

This case serves as an example of how a massive tumor can potentially get overlooked for many years, only to be detected unintentionally. The patient remained asymptomatic throughout the course of tumor growth, making this diagnosis particularly challenging. This case illustrates the importance of a comprehensive history and physical exam in all patients, especially those who are obese. The inherent biases some physicians may hold against obese patients may prevent the detection of potentially dangerous tumors, making timely diagnosis and management vital in improving patient outcomes.
